# Long-Term Determinants of Tuberculosis in the Ungulate Host Community of Doñana National Park

**DOI:** 10.3390/pathogens9060445

**Published:** 2020-06-05

**Authors:** Patricia Barroso, José A. Barasona, Pelayo Acevedo, Pablo Palencia, Francisco Carro, Juan José Negro, María José Torres, Christian Gortázar, Ramón C. Soriguer, Joaquín Vicente

**Affiliations:** 1 Instituto de Investigación en Recursos Cinegéticos (IREC) CSIC-UCLM-JCCM, 13071 Ciudad Real, Spain; pelayo.acevedo@uclm.es (P.A.); pablo.palencia@uclm.es (P.P.); christian.gortazar@uclm.es (C.G.); joaquin.vicente@uclm.es (J.V.); 2 VISAVET, Animal Health Department, Veterinary School, Complutense University of Madrid, 28040 Madrid, Spain; joseangel.barasona@gmail.com; 3 Estación Biológica Doñana, CSIC, 41092 Sevilla, Spain; pcarro@ebd.csic.es (F.C.); negro@ebd.csic.es (J.J.N.); soriguer@ebd.csic.es (R.C.S.); 4 Departamento de Microbiología, Universidad de Sevilla, 41009 Sevilla, Spain; mjtorres@us.es; 5 CIBER Epidemiología y Salud Pública (CIBERESP), 28029 Madrid, Spain; 6 Escuela Técnica Superior de Ingenieros Agrónomos, UCLM, 13071 Ciudad Real, Spain

**Keywords:** tuberculosis, long-term study, Doñana National Park, wild boar, deer, cattle, shared infections, wildlife-livestock interface

## Abstract

Animal tuberculosis (TB) is endemic in wild boar (*Sus scrofa*), red deer (*Cervus elaphus*), fallow deer (*Dama dama*) and cattle in south and central Spain. In order to clarify the processes that operate in the medium and long-term, we studied TB at the wildlife–livestock interface in Doñana National Park for 14 years (2006–2018) in relation to host density, stochastic factors (rainfall) and environmental features (e.g., aggregation points such as waterholes). Wild boar showed the highest prevalence of TB (76.7%), followed by red deer (42.5%), fallow deer (14.4%) and cattle (10.7%). We found evidence of relevant epidemiological processes which operate over the long-term and interact with host and community ecology. Interestingly, the effect of high wild boar population density on increased TB rates was mediated by sows, which could determine high incidence in young individuals already in maternal groups. Rainfall significantly determined a higher risk of TB in male red deer, probably mediated by sex-related differences in life history traits that determined more susceptibility and/or exposure in comparison to females. The positive association between the prevalence of TB in fallow deer and cattle may indicate significant interspecies transmission (in either direction) and/or similar exposure to risk factors mediated by ecological overlapping of grazing species. The identification of long-term drivers of TB provided evidence that its control in extensive pastoral systems can only be achieved by targeting all relevant hosts and integrating measures related to all the factors involved, such as: population abundance and the aggregation of wild and domestic ungulates, environmental exposure to mycobacteria, cattle testing and culling campaigns and adjustments of appropriate densities.

## 1. Introduction

A central problem concerning studies on the ecology of wildlife diseases is that many of the most important ecological, evolutionary and human-driven processes affecting host and pathogens may occur over multiple years or even decades. Many important questions, particularly for those pathogens maintained in complex host communities [1] can only be answered with data that extend over many years [2,3]. Long-term studies, therefore, provide the necessary temporal perspective to understand processes that operate over wide temporal scales [4]. Some advantages of long-term approaches in wildlife disease epidemiology are the possibility of carrying out: (i) analyses of density-dependent effects; (ii) analysis of stochastic factors; (iii) evaluations of time-delayed effects, including the effects within/among species; (iv) studies for detecting disease emergence patterns; (v) evaluation of epidemiological processes on multiple scales, from individual to population; (vi) analyses for detailing the role that social systems and between- and within-individual heterogeneity in infection transmission; (vii) studies that provide valuable information applicable to the management of sanitary and socio-economic problems; and (viii) assessments of risk management strategies, particularly, adaptive strategies [3,5,6]. However, despite the relevance of wildlife diseases to human and animal health, livestock productivity and conservation, long-term field studies of free-living wildlife hosts are relatively rare, and particularly, in multi-host systems taking place at the interface with livestock [3,6,7]. 

Animal tuberculosis (TB caused by *Mycobacterium tuberculosis* complex bacteria, MTC, including *M. bovis* and *M. caprae*) is a chronic infectious disease with a complex multi-host epidemiology, infecting many wildlife and livestock species [8]. It also supposes a zoonotic risk to humans, with relevance for public health. Mycobacteria have a long persistence in the environment, especially in humid and shady sites [9]. The infection spreads through direct and indirect routes, the latter being key for the inter-species transmission [10,11]. Indirect transmission usually occurs through shared resources such as water or food [12,13]. TB is endemic in cattle and wild ungulates in south and central Spain (SCS) [4,14]. Previous research concluded that ungulates, mainly Eurasian wild boar (*Sus scrofa*) and red deer (*Cervus elaphus*), are the wild reservoir hosts of TB in this area [4,15,16]. They can maintain and transmit the pathogen, even in the absence of other reservoir hosts [4,15,17]. The richness of ungulate host species, including cattle, correlates with increased community competence to maintain and transmit pathogens of the MTC in game-managed and wild areas in Mediterranean Spain [1].

In Doñana National Park (DNP, South West Spain), the wild ungulate community (including wild boar, red deer and fallow deer, *Dama dama*) sympatrically occurs with free-ranging cattle [18]. The prevalence rates of TB observed in DNP are among the highest reported in the literature, especially in wild boar [19]. Since the first case of TB was diagnosed [20], MTC has been isolated in the Iberian Lynx (*Lynx pardinus*), red fox (*Vulpes vulpes*) [21,22], red deer, fallow deer and wild boar [18,19,23,24,25], just as in cattle. There is also serological evidence of infection in badgers (*Meles meles*) [26]. Our research team has conducted studies on the prevalence of TB and infection routes in the wild ungulate community of DNP since 2006. Between 2006 and 2012, a sample of 570 wild boar, 190 red deer and 189 fallow deer was analysed, describing high prevalences [18,19,24,25]. Further samples were collected up to 2018, and there has been an attempt to test for MTC twice a year through the National Eradication Programme that removes all positive individuals [27]. Research in DNP has provided essential understanding on the epidemiology and ecology of TB in this diverse host community and such a complex environment [18,19,23,24,25]. Some of the previous findings regarding the spatial ecology of interactions at the wildlife–livestock interface [18,25,28,29,30], the population dynamics and the molecular epidemiology [14,23,24] are noteworthy. Research in DNP has also provided evidence of the relevance of some population and environmental factors in the transmission and maintenance of MTC [13]. However, these studies were carried out for relatively brief periods. Hence, a long-term perspective at the population level has been required to assess the impact on the TB epidemiology of these factors as well as other possible ones which operate on broader temporal scales, providing the basis for investigations on the role and factors determining pathogen rates, persistence and spread. 

In this context, using the data on ungulate surveillance in DNP for a 14-year period our aims were: (i) to evaluate the factors (individual, populational and environmental) modulating the prevalence of TB, and (ii) to assess the factors operating in the long-term (density-dependent and stochastic) in order to explain the temporal trend of the prevalence of TB in the host community of DNP from 2006 to 2018. 

## 2. Results

### 2.1. General

**Figure 1:**
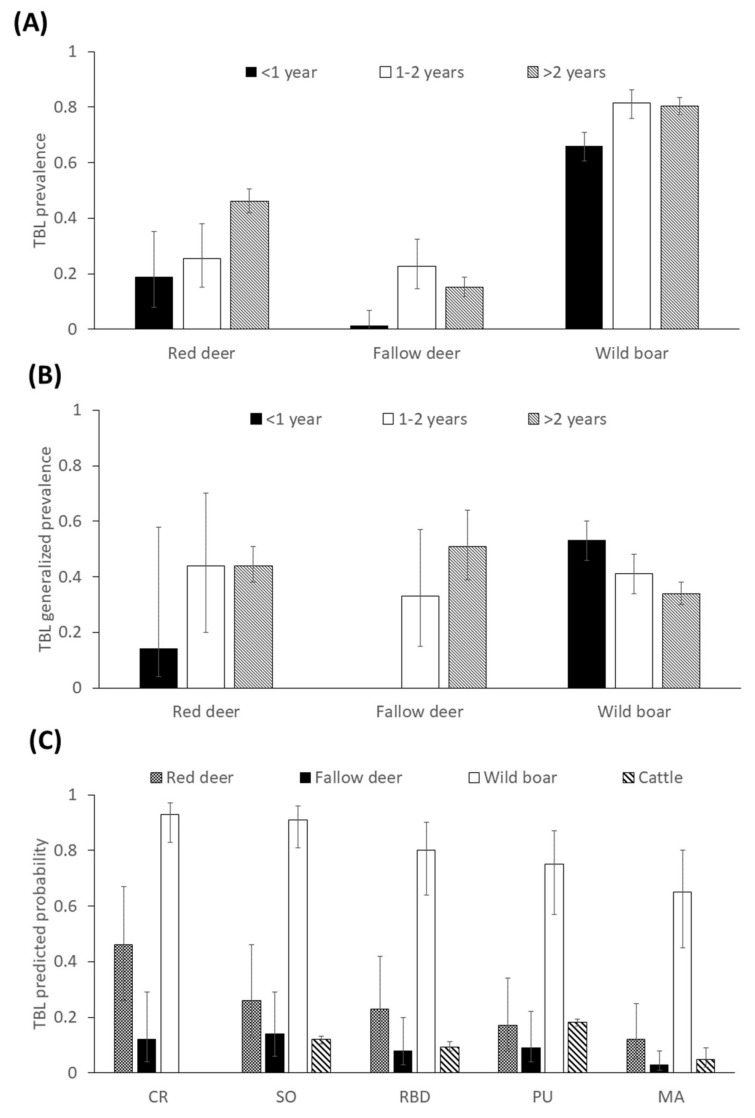
(**A**) Prevalence (±confidence interval (CI) 95%) of tuberculosis-like lesions (TBL) depending on age class in red deer, fallow deer and wild boar. (**B**) Generalized prevalence (±CI 95%) of TBL depending on age class in red deer, fallow deer and wild boar. (**C**) Predicted probability of TBL obtained from selected generalized linear mixed models (GzLMMs) (±CI 95%) for the respective species studied depending on the sampling area, from north to south areas (see Figure 11 below for a map of the areas with their full names).

The prevalence of TBL (±confidence interval (CI) 95%) in wild boar was 76.7 ± 2.5 (n = 1235; average annual prevalence (TBL ± CI 95%) of 77 ± 8.5; n = 14), followed by red deer (42.5 ± 4.7; n = 642; average annual prevalence of 42.3 ± 7.3; n = 14) and fallow deer (14.4 ± 2.6; n = 637; Figure 1A; average annual prevalence of 16.2 ± 5.5; n = 14), whereas the average annual incidence (±CI 95%) for cattle (based on the information from the skin testing campaign) was 10.7 ± 1.8 (n = 14). When considering TB-positive wild animals, fallow deer, red deer and wild boar presented comparable prevalence rates of generalized TBL (in at least two of the three anatomic locations studied), ranging from about 40–45% (46.7 ± 10.5, 43.6 ± 6 to 39.3 ± 3.1, respectively). Considering age classes, overall, increasing age trends were indicated by the prevalence of TBL (Figure 1A, statistical results are shown below). Generalized prevalence of TBL (Figure 1B) showed an increasing age pattern in both deer species, but apparently decreased with age in the wild boar. Spatially, contrasted prevalences of TBL were apparent among areas, showing a decreasing north to south gradient (Figure 1C; statistical results are presented below).

**Figure 2:**
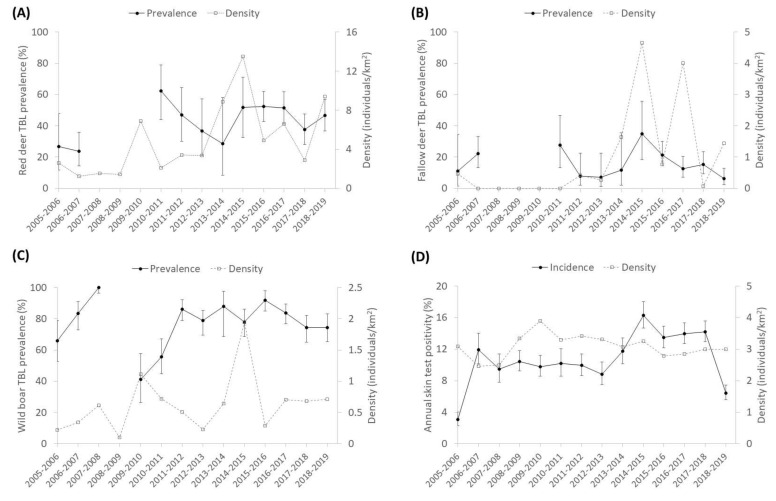
Temporal trend of the prevalence of tuberculosis-like lesions (TBL) (±CI 95%) and population density (individuals/km^2^) in (**A**) red deer, (**B**) fallow deer, (**C**) wild boar, and (**D**) annual skin test positivity (%) in cattle.

**Figure 3:**
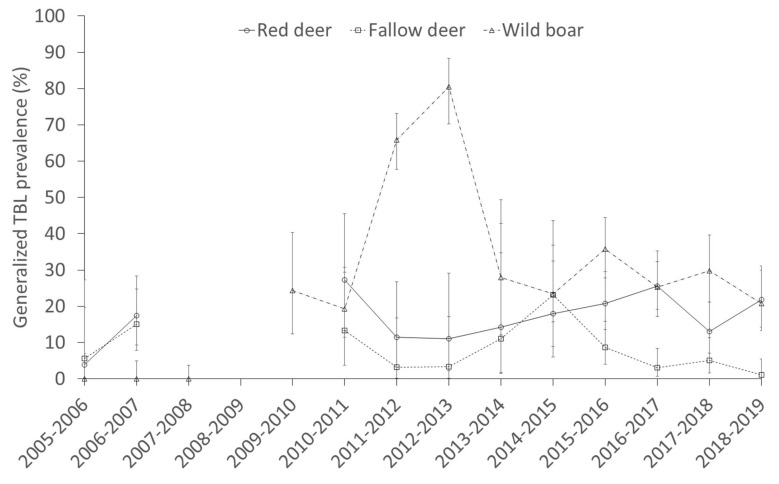
Temporal trend of the generalized prevalence (±CI 95%) of tuberculosis-like lesions (TBL) in red deer, fallow deer and wild boar.

**Figure 4:**
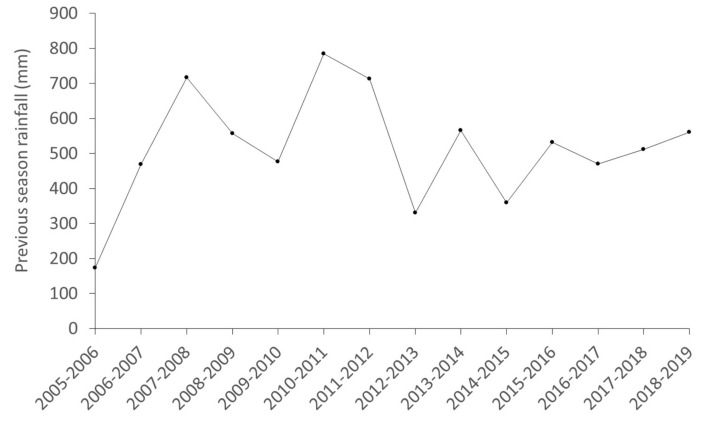
Temporal trend of the annual rainfall (mm).

As regards the temporal trends, Figure 2 and Figure 3 show the annual prevalence of TBL and generalized prevalence of TBL (%) and the population density (ind/km^2^) of wild ungulate species and cattle. The temporal trend of the previous season rainfall (mm) is also displayed (Figure 4). Overall, the prevalence of TBL showed marked annual fluctuations, especially in wild ungulates. It was remarkable that the prevalence of TBL increased in red deer over the study period compared with the initial situation (season 2005 to 2007), and that a marked decline for two years was observed in wild boar in 2009/10–11/11 (although no data were available in the 2007–2008 season) with a subsequent fast recovery to a very high prevalence. Since 2016–2017 a decline in the prevalence of TBL has been apparent in fallow deer and cattle, just as for the generalized presence of TBL in fallow deer and wild boar since 2015–2016 (Figure 3). 

### 2.2. Factors Determining the Presence of Tuberculosis-Like Lesions (TBL)

We selected the full models since no differences <2 cAIC values were observed when considering other potential models during a backward stepwise model selection procedure. Concerning the exploratory GzLMM on the presence of TBL, we found statistical differences among sampling areas for all the species (wild boar, *F* = 9.7, df = 1211, *p* ≤ 0.01; fallow deer, *F* = 3.4, df = 618, *p* ≤ 0.01; red deer, *F* = 7.1, df = 624, *p* ≤ 0.01; and cattle, *F* = 23.05, df = 34, *p* ≤ 0.01). The presence of TBL decreased from north to south (Figure 1C). This pattern was more marked in red deer and wild boar compared to cattle and fallow deer. 

Regarding the final GzLMMs on the presence of TBL, which incorporated other factors (previous season’s rainfall and annual ungulate community densities and prevalences of TBL), and controlled for the sampling area and season as random effects, results are shown in the Table 1, separately for each species.

As for individual factors, the age class was statistically significant for wild boar (younger individuals presented less TBL presence), it interacted with prevalence (see below), and sex significantly interacted with rainfall in red deer (see below). 

**Figure 5:**
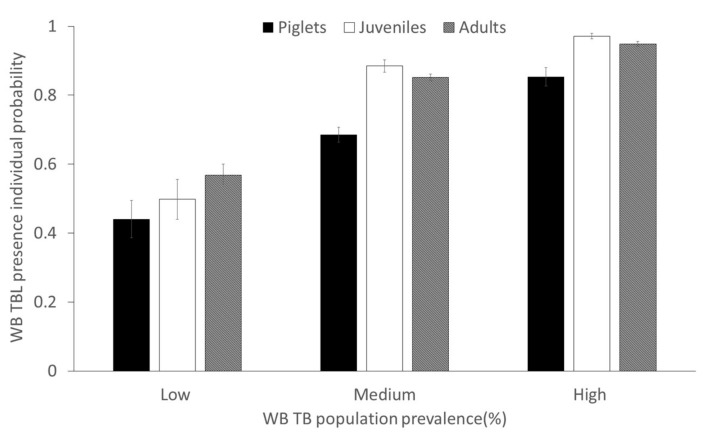
Predicted probability (±CI 95%) of the presence of tuberculosis-like lesions (TBL) in wild boar depending on the interaction between prevalence rates and age class. The three categories of prevalence considered based on 33 and 66 percentiles are: low (≤70%), medium (>70–≤93.8%) and high (>93.8%).

Regarding the explanatory factor for the prevalence of TBL host species, which can be considered an intra-specific risk for TB transmission, species-specific TBL annual prevalence was always a positive significant risk for all species and, as mentioned above, it significantly interacted with age in wild boar, so that the effect was more evident in individuals younger than 1 year old (Figure 5). 

**Figure 6:**
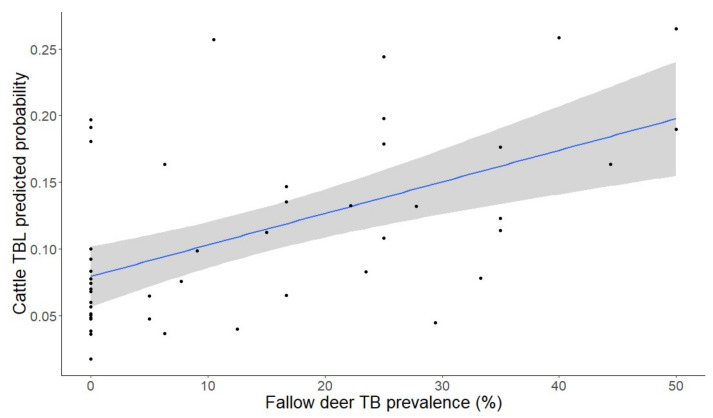
Predicted probability (±CI 95%) of the presence of tuberculosis-like lesions (TBL) in cattle depending on the prevalence of fallow deer TBL (%).

Regarding interspecific relationships, the prevalence of TBL in fallow deer was significantly (as explanatory) positively associated with the presence of cattle TBL (as response) (*F*_1,37_ = 1.16, *p* = 0.01; Figure 6).

**Figure 7:**
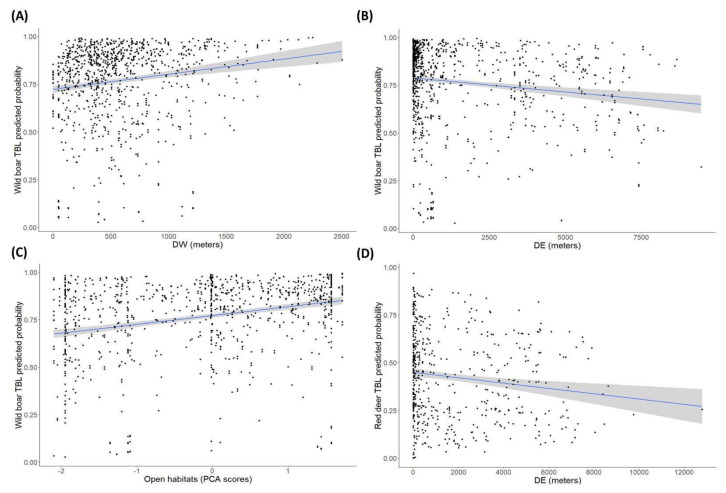
Predicted probability (±CI 95%) of the presence of tuberculosis-like lesions (TBL) in (**A**) wild boar depending on the distance to water bodies (m), (**B**) wild boar depending on the distance to the ecotone (m), (**C**) wild boar depending on the cover level of open habitats, measured according to the principal component analysis (PCA) scores from axis 1, and (**D**) red deer depending on the distance to the ecotone (m).

In relation to habitat factors, most of the significant effects were detected in wild boar. The further the distance to water bodies, the higher the presence of TBL (Figure 7A) was, whereas the closer to the ecotone (Figure 7B) and the higher availability of open habitats (Figure 7C), the higher the presence of TBL. Similarly, the proximity to the ecotone corresponded to statistically significant higher presence of TBL in red deer (Figure 7D).

As for density-dependent factors, no direct effects were found; however, statistical effects were revealed for generalized TBL (see below).

**Figure 8:**
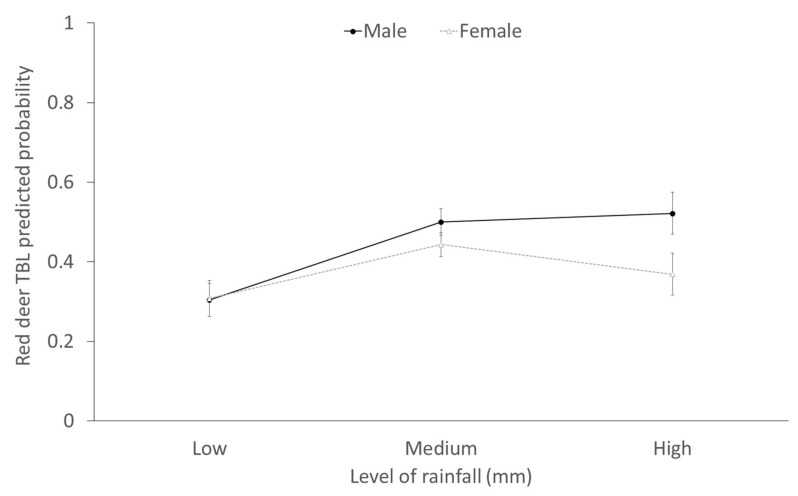
Predicted probability (±CI 95%) of the presence of TBL in red deer depending on the interaction between sex and rainfall. The three categories of rainfall considered based on 33 and 66 percentiles are: low (≤469.5 mm), medium (>469.5–≤560.9 mm) and high (>560.9 mm).

As mentioned above, rainfall (stochastic factor) significantly interacted with sex to explain the presence of TBL in red deer, so that a high annual rainfall is specifically associated with a higher presence of TBL in males (Figure 8).

### 2.3. Factors Determining the Presence of Generalized TBL 

**Figure 9:**
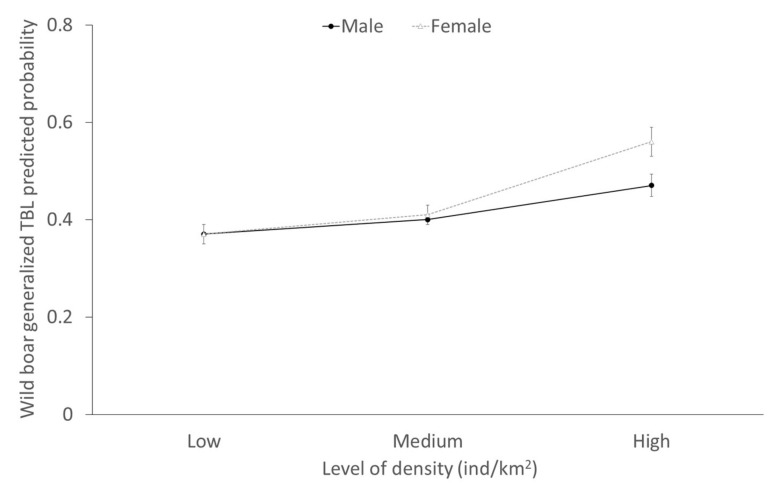
Predicted probability (± CI 95%) of the presence of generalized TBL in wild boar depending on the interaction between sex and density. The three categories of density considered based on 33 and 66 percentiles are: low (≤0.09 ind/km^2^), medium (>0.09–≤1.06 ind/km^2^) and high (>1.06 ind/km^2^).

Concerning the models of the individual presence of generalized TBL (Table 2), the annual specific prevalence of generalized TBL was positively associated in wild boar, fallow deer and red deer. The interaction between the density and sex was significant in wild boar and the positive association between high densities and the presence of generalized TBL in females was more evident compared to males (Figure 9). 

## 3. Discussion

This long-term study on TB in wildlife addresses a multi-host community including livestock, which implies added value among the scarce literature on shared wildlife-livestock infections. We found evidence of the potential effects exerted by stochastic and density-dependent factors on TBL, once controlled for other relevant drivers, which can only be assessed through a long-term perspective.

### 3.1. General Patterns of the Presence of TBL

**Figure 10:**
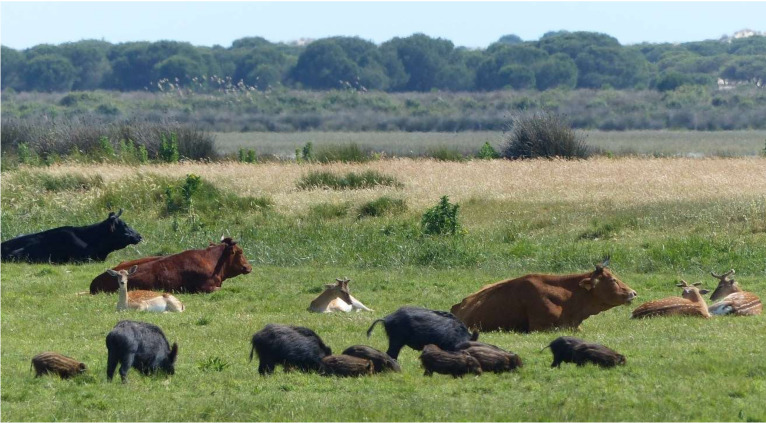
Cattle, wild boar and fallow deer rest and graze together in close proximity on the ecotone between marshland and scrubland in Doñana National Park. Habitat sharing among ungulate species facilitates the transmission of *Mycobacterium tuberculosis* complex bacteria (MTC) and explains why the eradication programme in cattle has been ineffective so far. Credit: Juan J. Negro.

The prevalences of TBL shown in this study were high compared to those obtained in similar studies on wildlife populations elsewhere [31,32,33,34]. The pattern of TBL presence associated with species, space and individual factors mostly confirms previous findings. Interestingly, the prevalence of TB in fallow deer associated with that of cattle, which is probably caused by the spatial overlap between these species (see, e.g., Figure 10). The common use of pastures, mainly at the “vera” ecotone (meadows) takes place especially during late summer (August–October), and this can be a relevant season for the spread of TB. This ecological overlap may lead to similar environmental TB drivers in both species. The role of fallow deer in the maintenance and transmission of TB to cattle is discussed below.

Overall, a growing temporal trend was observed in the annual incidence of TBL in cattle, showing rates above 10% in the majority of years within the study period, which indicates continuous re-infection and that the current TB control in cattle is ineffective in eradicating the infection in DNP [24]. We note that TBL figures in cattle are not comparable to those of wildlife since in the domestic species positive animals to the skin test are sacrificed each year and consequently, only “new infected” animals are identified in each campaign. The TBL figure, therefore, represents incidence (percentage of annual skin test positivity) and not prevalence, however in terms of evaluating the association of trends and evaluating risks in the long-term, they provide an useful approach.

The decline in the prevalence of TBL in wild boar that occurred as a result of the intense population control occurring in 2008–2009 that noticeably reduced wild boar densities is of particular note, and the subsequent speedy recovery of TBL levels after this population control was curtailed [35]. A high peak of generalized TBL in wild boar is evidenced thereafter (Figure 3). In systems that harbour virulent parasites, culling can reduce the prevalence of the disease [36], and in DNP wild boar culling temporally contributed to controlling TB, but efforts need to be maintained over time to have an effect on long-term TB dynamics.

**Figure 11:**
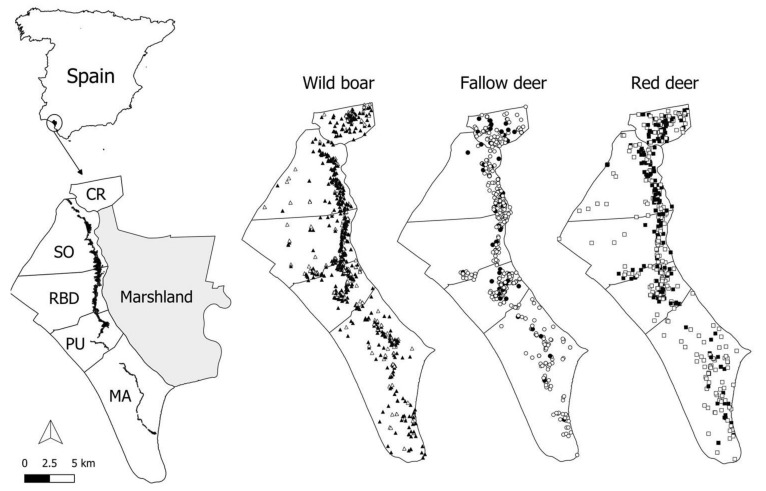
Map of the study area, Doñana National Park. The sampling areas (cattle management units: Coto del Rey (CR), Sotos (SO), Doñana Biological Reserva (RBD), Puntal (PU) and Marismillas (MA)) are delimited and the ecotone is displayed by a dark band. Sampled wild boar (triangles), fallow deer (circles) and red deer (squares) are shown. Black symbols mean animals positive for tuberculosis-like lesions.

Spatially, a north to south gradient in the prevalence of TBL was observed. This spatial pattern had already been noted previously [24]. However, a partial dilution of this spatial pattern is observed for all the species, indicating that the spread of TB in DNP is also modulated by increasing prevalences in less-affected areas of the south. The cattle population has increased in the southernmost part of the park during the last 5 years and, therefore, an effect also modulated by domestic populations cannot be discarded. Actually, the southern part of the area, called marshland in Figure 11, is not completely isolated from Marismillas (our southernmost study area) and cattle have been there for the last 3 years or more. This may exemplify the added sanitary risk if new cattle stocks were introduced in the areas of the park still free of cattle, as is insistently demanded by breeder associations. As recently shown by Barasona et al. [1], the concomitant effect of adding diversity and density of hosts (including domestic breeds) to the TB host community increases the community’s ability to maintain and transmit the pathogen. The similar spatial trend over time indicates that the MTC epidemiology in DNP partially responds to similar drivers across species, probably mediated by similar exposure patterns, i.e., environmental factors, but also cross-specific transmission. The lower prevalence observed in fallow deer reinforces the theory of the apparent lower natural host susceptibility of this species in comparison with other wild ungulates of the park, suggested in previous studies developed in DNP [24]. However, we cannot ignore differences in exposure in a grazing species that mainly use open areas, in contrast to the more habitat generalist wild boar and red deer. In DNP, intra and interspecific contact networks, and the subsequent molecular epidemiology pattern (distribution of the *M. bovis* types), seems to act at a very local scale at the host community level [19,25,30]. Previous studies on molecular epidemiology of TB in DNP have suggested that a local transmission frequently occurs between species [19]. 

As for individual factors, interestingly, no differences were observed in the proportions of generalized TBL among the hosts. However, this does not necessarily mean the severity and extension of lesions do not vary among species, as well as the level of mycobacteria excretion, which is very high for wild boar in DNP (over one third [37]), but still unknown for deer species in that area. In Portugal, red deer have been regarded as very relevant in terms of MTC shedding [38]. Concerning the age factor, the growing TBL prevalence pattern observed in wild boar has been frequently reported in the literature [24,39,40]. Interestingly, the high TB-induced mortality reported by Barasona et al. [41] in wild boar could explain the absence of an increasing pattern of generalized TBL with age, as observed in this species. Beyond prevalence comparisons and general patterns, we identified some factors that can exert an effect in the long-term, and that we discuss in the following section. 

### 3.2. The Environmental Features

Our results provide evidence that, for the main TB reservoir (in terms of high prevalence), the wild boar, the larger the distance to water bodies (straight-line distance to the nearest water hole, DW), the higher the presence of TBL (Figure 7A). Further, the closer to the ecotone (Figure 7B) and the greater availability of open habitats (Figure 7C), the higher the presence of TBL is. Similarly, the proximity to the ecotone corresponded to a higher presence of TBL in red deer (Figure 7D). Inter-specific transmission usually occurs by contaminated environmental elements such as pasture, water or mud [12,42]. Our results confirm that this indirect transmission in DNP takes place around aggregation points such as the pasture-rich ecotone and previous research indicates that this takes place especially during the dry season from June to September every year [18,29]. The ecotone between the marshland and shrublands offers high quality and palatable grasslands and shelter, especially during the dry seasons (summer and autumn) [43,44]. Previous studies where we modelled the spatial distribution of the ungulate community throughout DNP at a fine scale demonstrated that all ungulate species have a preference for the ecotone, where they spatially aggregate and interact [18,25,29]. In this context, the spread of MTC is favoured by higher intra and inter-specific contact rates as well as the ingestion of contaminated food or water [37,42]. 

Furthermore, wild boar sampled in areas with a higher availability of open habitats, especially grasslands, had a higher presence of TBL. According to previous studies, livestock–wildlife interactions are less frequent in areas with dense vegetation, considering that dense shrublands and woodlands constitute resting sites rather than foraging habitats for wild ungulates in DNP [25,29].

A large distance to water bodies implies that wild boar must traverse more distance to the nearest waterhole, i.e., there is scarceness of water points around, and this may exacerbate the use of the few water points available. Therefore, higher levels of aggregation are expected at these sites, and a subsequent increase in the risk of direct and/or indirect MTC exposure and transmission [10,40]. In this regard, previous studies demonstrated that the risk of TBL for wild ungulates was negatively associated with water point density in the surrounding area in Mediterranean habitats [10,18,45]. Specifically, Barasona et al. [18] showed a higher risk of TBL for wild boar and red deer in areas of DNP with lower local waterhole density. The same association was reported in Mediterranean areas and the USA [46] and dry sites in Africa [47,48], where cattle share not only water points and irrigated fields but also infections with wildlife. Waterholes are regarded as aggregation points for wildlife and livestock in which interactions are frequent and environmental maintenance of pathogens occurs, making them potential hotspots for MTC transmission [10,37,42]. 

As for density-dependence, the models on the individual presence of generalized TBL (Table 2) indicated that the interaction between density and sex was significant in wild boar, where the positive effect of density on the generalized TBL presence in females was more evident. Higher densities of wild boar originate increasing contact rates and depletion of resources, which favour exposure and, probably, are mediated by a reduced nutritional intake thereby increasing susceptibility [4]. The fact that these effects were more evident in females may be modulated by the physiological cost of reproduction (rearing of the piglet litter) during the dry season [49], which is the season when natural food availability is limited in Mediterranean habitats. This finding is very relevant since it may mediate the TB epidemiology from a very early age in wild boar. Research is needed comparing the situation in DNP with that in highly managed areas (i.e., fenced and year-round fed) for hunting purposes where the prevalence of TB in wild boar piglets is often low compared to that observed in adults [50]. Piglets already present very high prevalences of TBL in DNP, which may be mediated by direct transmission of MTC from sows, which develops into generalized TBL, severe lesions and subsequently increased excretion of mycobacteria in a density-dependent manner [4,16]. More research is needed to elucidate the potential mortality in juvenile wild boar due to TB, as suggested by the age decreasing pattern of generalized TBL (Figure 1B). 

### 3.3. Stochasticity: Rainfall

The effects of rainfall depended on the sex (significant interaction) to explain the presence of TBL in red deer, so that a high annual rainfall was more markedly associated with a higher presence of TBL in males. This suggests that in rainy years, greater exposure and/or susceptibility to TB may occur in males with respect to females. We speculate this may take place during the mating season. Very intense ruts (evidenced by increased aggressive interactions between stags) have been associated with higher food availability [51]. In Mediterranean ecosystems, the presence of food resources is key, considering that the rut occurs during a period of food scarcity [52]. In rainy years, many patches of grasslands remain available in the ecotone in DNP due to the high humidity, implying a higher aggregation of females and a maximum intrasexual competition for mating in males that is reflected in increased reproductive efforts. Furthermore, the effects of rainfall are not only mediated by water and food availability. In wet years the marshlands flood and ungulates remain aggregated on the ecotone, increasing the competition to mate in stags. The conflict between the immune response and the reproductive effort in red deer stags has been reported previously [53,54,55]. In this sense, the greatest investment during the rut (reproductive effort, testosterone metabolite levels and sexual signals) endanger the immunological defences, health status and fitness of red deer, making them more susceptible to infections [56,57,58]. Therefore, during the mating seasons of rainy years, the greater reproductive effort of stags may lead to a higher susceptibility to TB and to a higher interaction and more contact with other males. Both greater exposure and/or susceptibility to MTC infection by red deer stags in rainy years may, therefore, cause increased presence of TBL; however, our arguments remain speculative and more research is needed on this aspect.

No sex-dependent effects of rainfall were observed in the presence of TBL for fallow deer. For this species, the same immunosuppressant effect of reproductive efforts occurring during the rut season for fallow deer stags may take place [59]. However, the rut of fallow deer occurs later in Autumn and perhaps rainfall is more abundant and less determinant then. The fact that the prevalence of TBL is lower in fallow deer maybe also determine this absence of a relationship with rainfall [24].

### 3.4. Interspecific Relationships

The inter-specific interactions, as well as the intra-specific infection risks, are influenced by the ecological, behavioural and epidemiological factors typical of each species [39]. These factors such as scavenging or gregariousness can lead to greater intra-specific transmission [14]. Furthermore, individuals belonging to a particular species are exposed to the same risk factors in certain areas. Specifically, the annual intra-specific prevalence of TBL had a marked effect on the presence of TBL for wild boar, especially in piglets, which are a susceptible population in an “infectious environment” (family groups, environmental exposure). Our results also indicated a positive statistical relationship between the generalized intra-specific prevalence rates of TBL and the species-specific generalized presence of TBL for all wild species. Animals with generalized TBL become important super-shedders of MTC [38,60]. Hence, an increasing number of individuals with generalized TBL implies a higher excretion rate of mycobacteria by different routes [16] and subsequent increased direct and/or indirect transmission. 

Interestingly, we found a significant association between the presence of TBL in cattle and the prevalence of TBL in fallow deer (see Figure 6). Previous studies on spatiotemporal interaction patterns among wild ungulates and cattle carried out in DNP showed that the dynamics of TB transmission in this area is conditioned by environmental and habitat-related peculiarities which facilitate the spatiotemporal direct and indirect overlap between wildlife and livestock species [25,29,30] (Figure 10). Interestingly, high contact rates between fallow deer and cattle have been reported in DNP [13,30]. Both species share a preference for open habitats. In this sense, most studies have demonstrated that cattle tend to avoid shrublands in favour of open grassland and marshland just like fallow deer, which usually graze on the meadows on the periphery of the marsh and on the marsh itself [43,61]. Furthermore, the gregarious habits of both species, more marked in open habitats, favour these contacts, often establishing mixed groups [30,62,63]. This finding is in agreement with Triguero-Ocaña et al. [30] who recently suggested the potential substantial role of fallow deer in the maintenance and transmission of TB to livestock in DNP. In summary, the association of the presence of TBL between cattle and fallow deer is indicative of (i) sharing (in either direction) TB with cattle and/or (ii) being exposed to similar risk factors since both species are the closest ecologically speaking among the ruminants of the park (they are both grazers that prefer open lands, while red deer typically are mixed grazers/browsers).

## 4. Materials and Methods 

### 4.1. Study Area 

This study was performed in DNP, in southwestern Spain (37°09 N, 6°309 W), covering an area of 54,252 ha on the Atlantic coast. DNP is one of the most important natural reserves in Europe in terms of biodiversity, and it was declared a United Nations Educational, Scientific and Cultural Organization (UNESCO) Biosphere Reserve in 1980, expanding its boundaries in 2012. The eastern part of the park is taken up by seasonal marshland, whereas in the western part scrublands predominate in the north and sand dunes in the south (see Figure 11), and [25] for a more detailed description). There is an ecotone (a narrow strip of pastureland locally called “vera”) of high ecological richness between the marshland and scrubland [18]. This area of DNP has a dry, subhumid Mediterranean climate with marked seasonality. Mean annual precipitation is 550 mm (170 to 1000 mm) [64]. In the wet season (winter and spring) the marshlands may flood, but the ungulates can browse in some uncovered scrublands. Late summer conditions and their prolongation during autumn are the hardest season for the ungulates because of limited resources. The seasonal drought causes the aggregation of wild and domestic ungulates on the ecotone and around water sources in these seasons [25]. 

Human access to the park is restricted and it is managed by the competent conservation authorities in, the Autonomous Government of Andalusia [24]. Cattle and horse breeding are allowed inside the park [65]. Livestock populations include autochthonous and traditional breeds of cattle and horses such as the endangered breed “Marismeña”. Furthermore, horse breeding, aimed at promoting the recovery of “Caballos de las Retuertas”, occurs in some areas of Doñana National Park. The territory of DNP included in this study is divided into 5 livestock management areas: Coto del Rey (CR), Sotos (SO), Doñana Biological Reserve (RBD), Puntal (PU) and Marismillas (MA). Livestock is distributed through all the park, except in CR. In this area, located in the north of the DNP, cattle husbandry has not been allowed since 2002 as a conservation measure for the critically endangered Iberian lynx. Culling of the wild ungulate population is performed exclusively by park rangers as part of the park management scheme, and it is also used to carry out a health-monitoring programme [24,35]. 

### 4.2. Animal Sampling and Data Collection

From 2006 to 2018, 2594 ungulates including red deer (n = 642), fallow deer (n = 637) and wild boar (n = 1235) were randomly (sex, age class and health status were not selected) in autumn–early winter (October–January) shot by park rangers along with car drivers, and sampled as part of the DNP health-monitoring programme (approved by the Research Commission of DNP in accordance with the management rules established by the Autonomous Government of Andalusia. The geographical coordinates of the animals sampled were registered by portable GPS (Garmin Ltd., Olathe, KS, USA) (Figure 11).

The data collected included the sampling season (from 2006–2007 to 2018–2019), species, sex and age, which was determined by tooth eruption patterns [66,67,68]. Therefore, deer species were classified into three age classes: calves (<1-year-old), juveniles (1–2 years) and adults (≥3 years). Regarding wild boar, the categories were: piglets (<6 months), juveniles (6–24 months) and adults (>2 years). 

The sampling was performed according to European (EC Directive 86/609/EEC) and Spanish laws (RD 223/1988; RD 1021/2005), current guidelines for the ethical use of animals in research (ASAB, 2006), the Animal Experiment Committee of Castilla-La Mancha University and the Spanish Ethics Committee (PR-2015-03-08). Necropsies and sample collection were undertaken in the field by authorized veterinarians. The presence of tuberculosis-like lesions (TBL) was assessed and recorded by macroscopic inspection of the head, thoracic and mesenteric lymph nodes as well as abdominal and thoracic organs in the laboratory [15]. This analysis routinely included retropharyngeal and submandibular lymph nodes and tonsils in the head, tracheobronchial and mediastinal lymph nodes in the lungs and thorax and mesenteric lymph nodes, kidneys, liver and spleen in the abdomen. Gross lesions in other locations were also recorded. Lymph nodes were dissected, sectioned and carefully examined for gross lesions. Animals with TBL were classified as positive. When TBL are identified in at least two of the three anatomic locations analysed (head, thorax and abdomen) we considered the TBL as generalized, indicative of a more severe and evolved infection [40]. Concerning cattle, we compiled the information about the skin test campaign, in which single intradermal tuberculin test was conducted by veterinary authorities (19,869 tests) during the official TB control programme in DNP, which is performed annually. Through the positive rate (%) of the annual skin test campaign we determine the annual TB incidence in the cattle herds (newly infected animals divided by the total number of animals examined annually at each sampling site, which usually corresponds to the total cattle stock of the area, limited by DNP regulations). Positive animals are culled each year and consequently, only newly infected animals are identified in each annual campaign. The annual prevalence rates for wildlife and the annual incidence of TB in cattle were estimated for each cattle management area (CR, SO, RBD, PU and MA).

For each sampling season, the information concerning rainfall was collected from the meteorology station located at RBD [69]. The previous season’s rainfall was selected to be included in our models because of its potential relevance to population dynamics and effects on susceptibility or exposure to many pathogens in Mediterranean environments [4,70]. We established three categories of rainfall: low rainfall (≤470 mm), medium rainfall (>470–˂561 mm) and high rainfall (>561 mm) to display the results. To assess the effect of environmental factors on the risk of TBL, several variables were selected due to their influence on ungulate behaviour, distribution and epidemiology in DNP and other areas of SCS [4,25,43]. We used the same environmental variables that were selected in the study by Barasona et al. [18]. For that purpose, a grid of 100 × 100 m was created, generating territorial units in which we calculated: straight-line distance (m) to nearest water hole (DW); straight-line distance (m) to nearest marsh-shrub ecotone (DE); proportional cover of dense scrub (LT1); proportional cover of low-clear shrubland (LT2); proportional cover of herbaceous grassland (LT3); proportional cover of woodland (LT4); proportional cover of bare land (LT5); and the proportional cover of watercourse vegetation (LT6). This grid was merged with the exact location of each animal sampled using a point sampling tool with QGIS version 2.12.1 [71]. Landcover data was obtained from Andalusia Environmental Information [72]. 

A distance sampling methodology was used to monitor the population density of ungulates. Specifically, we sampled 7 line transects of 10–15 km distributed through the study area. For red deer and fallow deer, the surveys started two hours before sunset during September and were carried out from a vehicle (average speed was 10 km·h^−1^). For wild boar, we repeated the transect one hour after sunset in order to increase the sample size. During surveys, the distance from the observer to the animals was recorded with a telemeter. Distance Sampling 6.2 software was used to analyse the data [73]. Data were right-truncated when the probability of detection was lower than 0.15 [74]. Half-normal, uniform and hazard rate models for the detection function were fitted against the data using cosine, hermite polynomial, and simple polynomial adjustment terms fitted sequentially. The selection of the best model was based on Akaike’s information criterion (AIC) [75]. Stratified analysis was done. We defined three strata, according to abundance and visibility, of: shrubland, marshland and the ecotone. The data of all the years (2006–2018) were considered to estimate a detection function for each stratum, and the data of each strata, year and livestock management area were considered to estimate the encounter rate and group size. Finally, a density value for each year and livestock management area was estimated. 

### 4.3. Statistics

The presence of TBL per individual (as a binomial response variable) was related to a range of explanatory variables by using generalized linear mixed models (GzLMMs). Collinearity between environmental variables was explored previously and a principal component analysis (PCA) was performed to reduce the dimensions of the database in terms of environmental information. These variables were summarized in open habitats, in which LT3 predominates, and watercourse vegetation, in which LT6 predominates. 

Explanatory approach: in a first exploratory approach to study the statistical differences of the presence of TBL among sampling areas (CR, SO, RBD, PU and MA), we designed GzLMMs (the presence of TBL as a response variable) for each species (red deer, fallow deer, wild boar and cattle) where the sex and age classes, DW, DE, open habitats and watercourse vegetation were the explanatory variables. The sampling season was fitted in the model as a random-effect factor.

While the first preliminary approach aimed to provide the statistical basis to differences in the presence of TBL among study areas, the main approach of this study is to generalize the effect of the factors studied regardless of the sampling area. Therefore, the definitive models included sampling area as random factors. We then used the final models to evaluate the influence of the different determinants on the presence of TBL (as a response variable) separately for each species (red deer, fallow deer, wild boar and cattle). The explanatory variables were sex, age class, previous annual rainfall, wild ungulate population densities, cattle density, the prevalence of TBL in wild ungulates and incidence of cattle, DW, DE, open habitats and watercourse vegetation. The sampling area and season, as well as its interaction, were fitted in the models as random effect factors. The selection of the “best models” was performed using the corrected Akaike’s information criterion (cAIC) (Akaike, 1974). The assumptions of binomial GzLMMs were met in all the models [76]. We always used a binomial error and a logit link function. Significant *p*-value was set at 0.05. The predicted probability of TBL obtained from the models was used to represent results, which account for all the factors in the models. 

In order to explore a temporal pattern, cross-correlations and autocorrelations with the real prevalence of TBL and the predicted probability of TBL in the different species studied were used. With this purpose, we also introduced the average densities of the previous two and three years in further models.

The statistical analyses were done using IBM SPSS 19.0 software (IBM Corporation, Somers, NY, USA; [77]) and R software version 3.5.2 [78].

## 5. Conclusions

- The comprehensive monitoring carried out during the study period at the livestock–wildlife interface in DNP has provided evidence of relevant epidemiological processes which operate in the long term within the TB host community. The effects of the TB determinants are not straightforward but operate in complex interactions, often related to the ecology of the hosts.
  - The effect of wild boar density on TB epidemiology mediated by sows may determine a high incidence in maternal groups from a very early age.
  - Stochastic factors may determine the presence of TBL by interacting with the host’s natural life history, as evidenced in male red deer.
  - The association of the presence of TB in fallow deer with that of cattle may indicate interspecies transmission (in either direction) and similar exposure to risk factors mediated by ecological overlap (they both use the same pasture areas).
- In DNP, as well as in other protected areas, hunting is not allowed and there are no natural predators. Therefore, the health surveillance and population control of wild ungulates and comprehensive management of cattle stocks, including the application of the national TB programme, must be a priority to control the disease at the wildlife–livestock interface. Since increases in the diversity and density of the host community of TB increases the ability of the system to maintain and transmit the pathogen in Mediterranean assemblages, expanding the distribution of cattle over other areas of the park, as demanded by breeders, could have a negative effect on the distribution and rates of TB.
- This research illustrates that the understanding of the main determinants of the maintenance and spread of a pathogen in wildlife, livestock and human populations requires integrated wildlife monitoring. This includes the so called “denominator” (or population) information [79,80].
- This long-term study provides a deeper understanding of the main drivers of a shared pathogen, MTC, which may help government agencies to develop improved risk-management strategies. We encourage administrations to conduct long-term studies and integrated monitoring and also to consider the impact of this issue on public health.

## Figures and Tables

**Table 1:** Test statistics from the GzLMMs for the presence of tuberculosis-like lesions (TBL) related to sex, age class, straight-line distance to nearest water hole (DW), straight-line distance to nearest marsh-shrub ecotone (DE), the proportion of open habitats and watercourse vegetation, previous season’s rainfall, annual density of wild ungulates and cattle, annual prevalences of wild ungulates and interactions among them. The model was fitted using sampling season, sampling site and their interaction as random factors. Parameter estimates for the level of fixed factors were calculated using a reference value of 0 for the male level in the variable sex, for ≤1 year in the variable age class.

	Wild Boar			Fallow Deer			Red Deer		
	*F* df(x,y)	Estimate ± SE	*p*	*F* df(x,y)	Estimate ± SE	*p*	*F* df(x,y)	Estimate ± SE	*p*
**Sex ^1^**	1.64 (1,1119)	Female: 1.17 ± 0.69	0.20	0.12 (1,604)	Female: −0.30 ± 1.88	0.73	1.64 (1,612)	Female: 2.25 ± 1.62	0.20
**Age ^2^**	3.88 (2,1199)	2 years: 2.79 ± 1.32 ≥3 years: 2.2 ± 1.01	**0.02**	0.04 (2,604)	2 years: 1.27 ± 4.43 ≥3 years: 1.47 ± 4.25	0.96	0.15 (2,612)	2 years: 2.24 ± 2.85 ≥3 years: 1.44 ± 2.66	0.86
**DW**	3.66 (1,1199)	0 ± 0	**0.05**	0.51 (1,604)	0 ± 0	0.48	0.08 (1,612)	−0 ± 0	0.78
**DE**	6.10 (1,1199)	−0 ± 0	**0.01**	0.80 (1,604)	−0 ± 0	0.37	5.24 (1,612)	−0 ± 0	**0.02**
**Open habitats**	12.21 (1,1199)	0.26 ± 0.08	**<0.01**	0.61 (1,604)	0.09 ± 0.12	0.44	2.40 (1,612)	0.11 ± 0.07	0.12
**Watercourse vegetation**	0.24 (1,1199)	−0.04 ± 0.08	0.63	1.68 (1,604)	−0.15 ± 0.11	0.20	0.09 (1,612)	−0.03 ± 0.1	0.76
**Previous Rainfall**	0.23 (1,1199)	−0 ± 0	0.63	0.09 (1,604)	0 ± 0.01	0.77	0.09 (1,612)	0 ± 0	0.76
**Red deer density**	0.04 (1,1199)	−0.01 ± 0.03	0.84	0.14 (1,604)	−0.02 ± 0.04	0.70	1.79 (1,612)	−0.36 ± 0.26	0.18
**Fallow deer density**	0.34 (1,1199)	0.04 ± 0.06	0.56	0.26 (1,604)	−0.24 ± 0.57	0.61	0.02 (1,612)	0.01 ± 0.04	0.88
**Wild boar density**	3.23 (1,1199)	−1.24 ± 0.68	**0.07**	0.26 (1,604)	0.11 ± 0.22	0.61	0 (1,612)	0.002 ± 0.17	0.99
**Prevalence of red deer TBL**	1.61 (1,1199)	−0.01 ± 0.01	0.20	0.05 (1,604)	0 ± 0	0.82	20.85 (1,612)	0.05 ± 0.03	**<0.01**
**Prevalence of fallow deer TBL**	0.30 (1,1199)	0.004 ± 0.01	0.59	7.03 (1,604)	0.01 ± 0.05	<0.01	0 (1,612)	−0 ± 0.01	0.97
**Prevalence of wild boar TBL**	85.18 (1,1199)	0.04 ± 0.01	**<0.01**	0 (1,604)	−0 ± 0.01	0.98	0.04 (1,612)	−0 ± 0.01	0.84
Sex*age	1.65 (2,1199)	Female* 2 years: −0.91 ± 0.51 Female* ≥3 years: −0.16 ± 0.36	0.19	0.20 (2,604)	Female* 2 years: −0.11 ± 1.43 Female* ≥3 years: −0.51 ± 1.31	0.82	0.75 (2,612)	Female* 2 years: −1.72 ± 1.52 Female* ≥3 years: −0.99 ± 1.35	0.47
Rainfall*sex	2.47 (1,1199)	Rainfall*Female: −0 ± 0	0.12	0.29 (1,604)	Rainfall*Female: 0.001 ± 0.003	0.59	3.90 (1,612)	Rainfall*Female: −0 ± 0	**0.05**
Density*sex	0.06 (1,1199)	Density*Female −0.04 ± 0.16	0.81	0 (1,604)	Density*Female −0 ± 0.11	0.99	0.602 (1,612)	Density*Female 0.03 ± 0.04	0.44
Rainfall*age	1.67 (2,1199)	Rainfall* 2 years: 0 ± 0 Rainfall* ≥3 years: 0 ± 0	0.19	0.04 (2,604)	Rainfall* 2 years: −0 ± 0.01 Rainfall* ≥3 years: −0 ± 0.01	0.96	0.67 (2,612)	Rainfall* 2 years: −0 ± 0.01 Rainfall* ≥3 years: −0 ± 0	0.51
Density*age	0.03 (2,1199)	Density* 2 years: 0.03 ± 0.26 Density* ≥3 years: 0.05 ± 0.18	0.97	0 (2,604)	Density* 2 years: 0.02 ± 0.4 Density* ≥3 years: 0.02 ± 0.36	0.99	2.24 (2,612)	Density* 2 years: 0.12 ± 0.27 Density* ≥3 years: 0.33 ± 0.23	0.11
Prevalence*age	6.09 (2,119)	Prevalence* 2 years: 0.04 ± 0.01 Prevalence* ≥3 years: 0.03 ± 0.01	**<0.01**	1.91 (2,604)	Prevalence* 2 years: 0.11 ± 0.05 Prevalence* ≥3 years: 0.08 ± 0.05	0.15	0.66 (2,612)	Prevalence* 2 years: 0.02 ± 0.03 Prevalence* ≥3 years: −0.01 ± 0	0.52
Rainfall*density species studied	2.58 (1,119)	0 ± 0	0.11	0.23 (1,60)	0 ± 0	0.63	0.01 (1,61)	0 ± 0	0.91

^1^ Reference value for sex: male, ^2^ Reference value for age: ≤1 year, * represents interactions among explanatory variables.

**Table 2:** Test statistics from the GzLMMs for the presence of generalized tuberculosis-like lesions (TBL) related to sex, age class, straight-line distance (m) to the nearest water hole (DW), straight-line distance (m) to the nearest marsh-shrub ecotone (DE), the proportion of open habitats and watercourse vegetation, the previous season’s rainfall, annual densities of wild ungulates and cattle, annual prevalences of wild ungulates and the interaction between sex and age, rainfall and sex, density and sex, rainfall and age, density and age, rainfall and density of the species studied. The model was fitted using sampling season, sampling site and their interaction as random factors. Parameter estimates for the level of fixed factors were calculated using a reference value of 0 for the male level in the variable sex, for ≤1 year in the variable age class.

	Wild Boar			Fallow Deer			Red Deer		
	*F* df(x,y)	Estimate ± SE	*p*	*F* df(x,y)	Estimate ± SE	*p*	*F* df(x,y)	Estimate ± SE	*p*
**Sex ^1^**	1.11 (1,800)	Female: −0.84 ± 1.06	0.29	1.93 (1,68)	Female: −8.35 ± 5.95	0.17	0.004 (1,246)	Female: −14.7 ± 210.89	0.95
**Age ^2^**	0.48 (2,800)	2 years: −0.59 ± 1.46 ≥3 years: −1.14 ± 1.25	0.62	0.55 (1,68)	2 years: 7.05 ± 324.75 ≥3 years: 1.90 ± 324.80	0.46	0.09 (2,246)	2 years: −175.87 ± 613.73 ≥3 years: −177.09 ± 613.73	0.91
**DW**	1.76 (1,800)	−0 ± 0	0.19	0.13 (1,68)	0 ± 0	0.72	1.16 (1,246)	−0 ± 0	0.28
**DE**	1.14 (1,800)	−0 ± 0	0.29	0.81 (1,68)	0 ± 0	0.37	0.007 (1,246)	0 ± 0	0.93
**Open habitats**	1.76 (1,800)	−0.14 ± 0.1	0.19	0.08 (1,68)	0.09 ± 0.31	0.78	0.557 (1,246)	0.1 ± 0.14	0.48
**Watercourse vegetation**	2.86 (1,800)	−0.17 ± 0.1	0.09	0.10 (1,68)	−0.11 ± 0.34	0.75	0.001 (1,246)	0.02 ± 0.15	0.91
**Previous rainfall**	0.38 (1,800)	−0 ± 0	0.54	0.69 (1,68)	−0.01 ± 0.01	0.41	0.102 (1,246)	−0.32 ± 1.02	0.75
**Red deer density**	0.60 (1,800)	0 ± 0.04	0.44	0.04 (1,68)	0.03 ± 0.13	0.84	0.095 (1,246)	−7.47 ± 23.16	0.76
**Fallow deer density**	0.26 (1,800)	−0.04 ± 0.08	0.61	0.60 (1,68)	2.52 ± 2.21	0.44	0.447 (1,246)	−0.09 ± 0.08	0.27
**Wild boar density**	0.38 (1,800)	0.28 ± 0.99	0.54	0.02 (1,68)	0.09 ± 0.73	0.90	0.426 (1,246)	−0.20 ± 0.28	0.47
**Bovine Density**	0.31 (1,800)	−0.07 ± 0.13	0.58	0.26 (1,68)	0.15 ± 0.29	0.61	0.044 (1,246)	0.01 ± 0.11	0.60
**Red deer generalized prevalence of TBL**	0.06 (1,800)	−0.01 ± 0.02	0.80	0.50 (1,68)	−0.03 ± 0.04	0.49	6.13 (1,246)	0.05 ± 0.02	**0.01**
**Fallow deer generalized prevalence of TBL**	2.72 (1,800)	−0.02 ± 0.01	0.10	6.05 (1,68)	0.07 ± 0.03	**0.02**	0.15 (1,246)	−0 ± 0.01	0.70
**Wild boar generalized prevalence of TBL**	59.74 (1,800)	0.07 ± 0.01	**<0.01**	0.05 (1,68)	−0.01 ± 0.03	0.82	0.33 (1,246)	−0.01 ± 0.01	0.57
Sex*age	0.44 (2,800)	Female* 2 years: −0.51 ± 0.57 Female* ≥3 years: −0.17 ± 0.45	0.65	0.22 (2,68)	Female* ≥3 years: 0.80 ± 1.69	0.64	0.25 (2,246)	Female* 2 years: −14.85 ± 210.89 Female* ≥3 years: 15.68 ± 210.88	0.78
Rainfall*sex	0.27 (1,800)	Rainfall* Female: 0 ± 0	0.60	1.80 (1,68)	Rainfall* Female 0.02 ± 0.01	0.19	0.04 (1,246)	Rainfall* Female: −0 ± 0	0.84
Density*sex	4.13 (1,800)	Density* Female 0.37 ± 0.18	**0.04**	0.50 (1,68)	Density* Female −0.30 ± 0.42	0.48	1.14 (1,246)	Density* Female −0.07 ± 0.06	0.29
Rainfall*age	0.27 (2,800)	Rainfall* 2 years: 0 ± 0 Rainfall* ≥3 years: 0 ± 0	0.60	1.37 (2,68)	Rainfall* ≥3 years: 0.01 ± 0.01	0.25	0.17 (2,246)	Rainfall* 2 years: 0.33 ± 1.02 Rainfall*≥3 years: 0.33 ± 1.02	0.84
Density*age	0.55 (2,800)	Density* 2 years: 0.28 ± 0.27 Density* ≥3 years: 0.15 ± 0.22	0.58	2.89 (2,68)	Density* ≥3 years: −1.97 ± 1.16	0.09	0.06 (2,246)	Density* 2 years: 7.75 ± 23.2 Density* ≥3 years: 7.75 ± 23.2	0.94
Rainfall*density species studied	0.31 (1,800)	−0 ± 0	0.58	0.17 (1,68)	−0 ± 0	0.68	1.47 (1,246)	−0 ± 0	0.23

^1^ Reference value for sex: male, ^2^ Reference value for age: ≤1 year, * represents interactions among explanatory variables.
